# Infections on the move: how transient phases of host movement influence disease spread

**DOI:** 10.1098/rspb.2017.1807

**Published:** 2017-12-20

**Authors:** D. R. Daversa, A. Fenton, A. I. Dell, T. W. J. Garner, A. Manica

**Affiliations:** 1 Institute of Integrative Biology, Biosciences Building, University of Liverpool, Crown Street, Liverpool L69 7ZB, UK; 2 National Great Rivers Research and Education Centre (NGRREC), East Alton, IL 62024, USA; 3 Department of Biology, Washington University in St Louis, 1 Brookings Dr, St Louis, MO 63130, USA; 4 Institute of Zoology, Zoological Society of London, Regents Park, London NW1 4RY, UK; 5 Department of Zoology, University of Cambridge, Downing Street, Cambridge CB2 3EJ, UK

**Keywords:** epidemiology, movement, spatial modelling, metapopulations, networks, host–parasite interactions

## Abstract

Animal movement impacts the spread of human and wildlife diseases, and there is significant interest in understanding the role of migrations, biological invasions and other wildlife movements in spatial infection dynamics. However, the influence of processes acting on infections during transient phases of host movement is poorly understood. We propose a conceptual framework that explicitly considers infection dynamics during transient phases of host movement to better predict infection spread through spatial host networks. Accounting for host transient movement captures key processes that occur while hosts move between locations, which together determine the rate at which hosts spread infections through networks. We review theoretical and empirical studies of host movement and infection spread, highlighting the multiple factors that impact the infection status of hosts. We then outline characteristics of hosts, parasites and the environment that influence these dynamics. Recent technological advances provide disease ecologists unprecedented ability to track the fine-scale movement of organisms. These, in conjunction with experimental testing of the factors driving infection dynamics during host movement, can inform models of infection spread based on constituent biological processes.

## 1. Introduction

Understanding how infectious diseases spread through spatial networks of hosts has been called a ‘holy grail' of epidemiology [1]. Spatial host networks portray host populations as a set of nodes in which hosts reside, and host movement among those locations serves as the links (i.e. edges) connecting the network [2,3]. As most disease-causing parasites cannot actively disperse, host movement also provides critical links for parasite infections to spread [2]. Characterizing these links is not straightforward, however. Multiple processes act on hosts during movement across the landscape that potentially influence infections. Dispersal ecologists refer to this period of movement after organisms depart a discrete location (e.g. household, habitat patch), but before arriving to a different location, as the transient phase [4]. Explicitly considering transient movement phases has provided a deeper understanding of the causes and consequences of wildlife movement [4], but this phase has largely been ignored in studies of disease spread.

Moving hosts are subject to changes in biotic and abiotic conditions that alter existing infections [5], cause mortality [6,7] or facilitate acquisition of new infections [8,9]. The infection status of individuals arriving into new locations may therefore be indirectly or unrelated to their infection status when movement is initiated. Here, we review the limitations of current approaches to studying infection spread and emphasize the benefits of explicitly considering the processes that occur during transient phases of host movement (hereafter referred to as ‘host transience'). First, we overview the existing methods examining the link between host movement and infection spread. Second, we propose a modelling framework that explicitly considers host movement and infection dynamics during transient phases, before developing testable hypotheses about the importance of factors influencing infection dynamics during host transience. We conclude by discussing how our framework can guide future research testing the role of host transience in the spatio-temporal dynamics of wildlife and human disease.

## 2. Current approaches for investigating the link between host movement and infection spread

Most research has focused on seasonal host migrations [5,7], but we broaden this perspective to consider any movement that connects spatially discrete resident locations of hosts. This includes large-scale seasonal migrations between breeding and non-breeding habitats, but also routine, local movements within populations (e.g. foraging between resource patches, mate searching among subgroups) or more regionally between different populations (e.g. dispersal). This definition of movement aligns well with existing spatial network frameworks and permits comparisons of infection dynamics during host transience at various scales.

### (a) Theoretical studies

**Figure 1.**
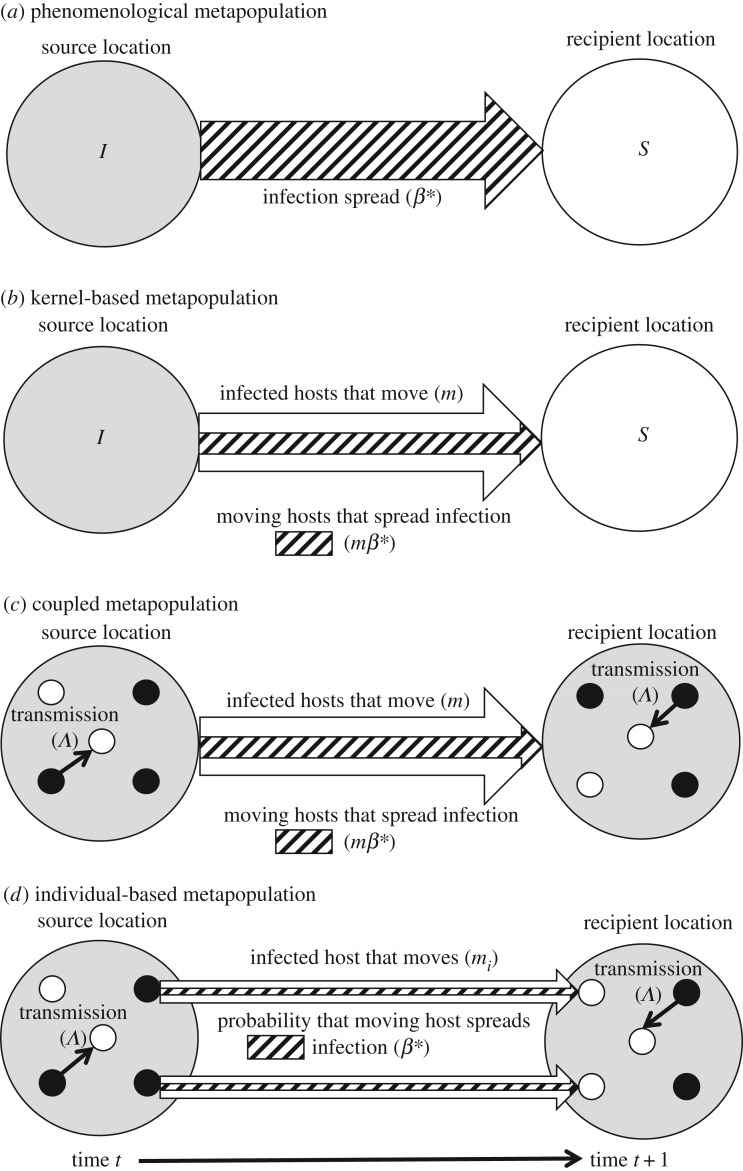
Metapopulation-based spatial disease models track locations of hosts and either (*a*) simulate infection spread based on connectivity measures without explicitly considering host movement or (*b*) define proportion of hosts that change locations between time steps (white arrow) with infection spread occurring from a proportion of hosts that change from infected locations to susceptible locations (striped arrow). (*c*) *Coupled metapopulation models* link local processes such as transmission (thin black arrow) to the between-location processes of host movement and infection spread. (*d*) *Individual-based network models* track movements of each host (denoted by subscripts *i*,*j*).

Spatial network models specify the geographical locations of hosts and their infections over time [3,10]. We define four broad categories of models describing the spatial dynamics of infection spread (figure 1), with some examples of each type provided in electronic supplementary material, table S1. Many existing spatial network models use metapopulation approaches [10], where the unit of measurement is the resident location rather than the individual, each with standard epidemiological states (e.g. susceptible, exposed, infected and recovered). The simplest versions are *phenomenological metapopulation models* (figure 1*a*) [11], which do not explicitly parameterize host movement, but instead model connectivity of groups, with rates of spread determined by physical processes, such as gravitation [12], percolation [13] and radiation [14]. Despite their simplicity, phenomenological models have accurately reproduced patterns of disease spread in human and wildlife populations. For example, the spread of plague in populations of great gerbils (*Rhombomys opimus*) occurs between resident locations (burrows) that are in closest proximity to one another [13], while the spread of influenza in humans is explained by the proximity and size of resident locations, with larger locations experiencing increased host movement and higher rates of infection [15]. *Kernel-based metapopulation models* (figure 1*b*) extend these models by including an explicit parameter for host movement (the mobility kernel, *m* [16]) that specifies a proportion of hosts that change locations between time steps. The rate at which infections spread to susceptible nodes (*S*) is a function of the mobility kernel, the number of infected nodes (*I*) and the probability that each movement successfully spreads the infection (β*):2.1
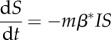
and2.2
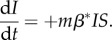

Kernel-based metapopulation models have seen widespread application in disease ecology and have been extended to consider effects of habitat quality in resident locations [17,18], host phenotypic variation [19] and the presence of alternative hosts [20]. Simpler models assume a fixed rate of movement between locations [11], or in proportion to the density of hosts in source locations [21]. However, Levy or random walks that characterize heterogeneities in movement trajectories of individuals are increasingly applied [22]. *Coupled metapopulation models* (figure 1*c*) incorporate within-location infection dynamics (e.g. transmission, recovery, births and deaths), and link these to the between-location dynamics of host movement (*m*) and infection spread (β*IS) [23]. Finally, while kernel-based and coupled metapopulation models track cohorts of hosts that move over time, *individual-based (or agent-based) metapopulation models* (figure 1*d*) have nodes that represent individuals, permitting tracking of the movement and transmission of each individual host [24]. Individual-based metapopulation models may uphold assumptions of homogeneous mixing within locations [25], though some agent-based models explicitly account for heterogeneous contact rates within locations [26].

While many models do explicitly account for host movement, infection spread *per se* is generally described in much simpler terms, typically as a constant probability of infected hosts spreading infection (β*). This simplification overlooks the potential for infections to be acquired [1,12] or lost [11,21], or for hosts to die [27] while moving. Although models may accurately reproduce spatial patterns of infection, ignoring the underlying mechanisms driving those patterns does not allow extrapolation to predict disease spread under novel environmental scenarios. In subsequent sections, we consider the consequences of relaxing these constraints.

### (b) Empirical studies

Owing to the difficulty in determining the location and infection status of moving hosts, many empirical approaches, such as mark–recapture (MR) surveys and genetic analyses (electronic supplementary material, table S2), infer movement and infection spread from data collected at resident locations. Ultimately, the lack of information on host transience poses limitations that cannot be overcome without additional approaches. For example, MR surveys of cliff swallows (*Petrochelidon pyrrhonota*) showed that prevalence of parasites in swallow colonies rose with increased arrivals by non-residents. However, colonies with the highest prevalence were also those with the most nests [28], highlighting how the contribution of movement to infection spread is difficult to disentangle from within-location factors solely through MR. Correlations between host arrival rates and prevalence may also reflect increases in susceptible hosts if many hosts are uninfected upon arrival [29]. Studies have also found weak [9] and even negative associations between host arrival and infection prevalence, for example after fish migrations [30].

Population genetics has revealed congruent patterns of gene flow between hosts and parasites. These overlaps, which have been found for parasites of both humans [31,32] and wildlife (reviewed by Mazé-Guilmo *et al*. [33]), are considered as evidence of the link between infection spread and host movement. Sampling of rapidly evolving RNA viruses, which have short generation times relative to the rate of host movement [34,35], has improved the temporal scale at which genetic analyses can focus. Streicker *et al*. [35] used this approach to reconstruct the recent spread of rabies in populations of vampire bats (*Desmodus rotundus*), and higher rates of viral gene flow than maternally inherited bat genes suggested male biases in spread. Whereas the above techniques cannot distinguish individual movements, Bayesian assignment tests, which use host and parasite genotypes, allow for individual-based assessments of host movement between resident locations [36]. Assignment tests have also proved useful for determining how landscape features affect infection spread by impeding host movement [36], but this technique is error prone [37]. Any genetic approach cannot reconstruct the path travelled by, and infection status of, hosts during transience.

Biologging techniques, such as radio telemetry and GPS tags, can overcome these issues by providing a more complete picture of host movement [38]. Craft *et al*. [19] used GPS devices on nomadic and territorial lions (*Panthera leo*) in a spatial network of prides in the Serengeti, which provided data for disease simulations that explicitly included host transience. Other biologging studies linked GPS locations to environmental data to assess effects of elevation [39] and landscape structure [26] on infection spread. A key challenge of biologging is acquiring infection data from hosts in transience. Capturing hosts to obtain samples may be dangerous and disrupt natural movement behaviours. As a result, remote tracking has provided detailed empirical data for modelling host movement in host networks, but infection spread must be inferred [19]. In addition, remote tracking is feasible for relatively few wildlife host-parasite systems, and remains costly.

The long distances travelled by many migratory hosts allow researchers to survey infections in hosts along different points in the migratory route, which perhaps has provided the most insight into infection dynamics during host transience (electronic supplementary material, table S2). Positive associations between host migration and spatial expansion of infections have been reported [40]. However, reduced infection prevalence among migrating animals has also been widely observed [7,30] (electronic supplementary material, table S2), possibly due to increased mortality of infected hosts [7], avoidance of infection through ‘migratory escape' [7] or recovery from infection while moving [5]) (see §4 for further discussion). Direct quantification of any of these processes in the wild is currently lacking.

## 3. Framework for integrating host transience into spatial network models of infection spread

**Figure 2.**
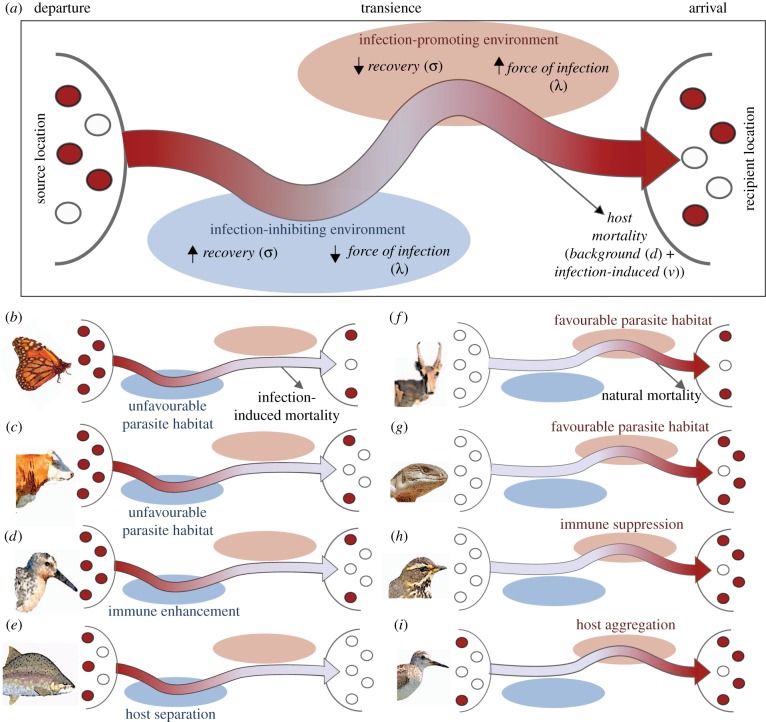
(*a*) Framework for capturing transient phase infection dynamics. The movement path of hosts and their infections (intensity/probability represented by darker shading of the arrow being higher intensity/probability) are categorized into three phases: departure, transience and arrival. During transience, infections are lost/reduced through background or disease-induced mortality of infected hosts, or as conditions during transience decrease exposure and/or cause deterioration of infections (i.e. recovery). Mechanisms that drive recovery include: (*b–c*) movement through habitats unsuitable for infections, which may occur with protozoal infections during monarch butterfly migrations [6] and with tick infections during ranging movements of livestock [41]; (*d*) enhancement of immune function during periods of movement, which may occur in migratory red knots [42]; and (*e*) dispersion of hosts that reduces contact, as evidenced by sea lice infections in migratory pink salmon [43]. Mechanisms that increase the force of infection during transience include: (*g–f*) movement through habitats with viable infective stages, which occurs with parasitic nematodes in migratory saiga [8] and dispersing pygmy blue tongue lizards [9]; (*h*) immunosuppression, such as the proliferation of latent bacterial infections in migratory redwing thrushes [44]; and (*i*) host aggregation, which occurs with avian influenza virus (AIV) infections during stopovers by migrating sandpipers [45]. (Online version in colour.)

To better understand how transient phases of host movement factor into spatial infection dynamics, we propose a framework that integrates concepts from dispersal ecology and spatial disease modelling (figure 2*a*). We conceptualize our framework as an individual-based metapopulation, but it could be applied to any of the spatial network models shown in figure 1. Briefly, host movement between spatially discrete locations is broken into three phases: departure, transience and arrival. While in transience, hosts can acquire infections (transmission) or recover from infections (recovery), and all hosts are subject to mortality, potentially at different rates for infected and uninfected hosts.

To illustrate mathematically the effect of these processes on host and infection dynamics, and the factors affecting them, we describe the dynamics of a cohort of moving hosts of size *M*, comprising *I* infected hosts and *S* uninfected hosts (*M = S + I*). Here, we used a simple host–microparasite framework [46], which ignores the infection load of hosts, for ease of illustration. More complex, tailored models could be developed as required. Host and infection dynamics during the transient phase can be described by3.1
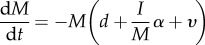
and3.2
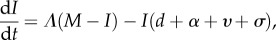
where *d* is the background host mortality rate, *α* is the parasite-induced host mortality rate, 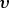 is the host arrival rate at the recipient location (i.e. 1/duration spent moving) and *σ* is the host recovery rate from infection (for simplicity here, we assumed infected hosts recover to be susceptible to reinfection, but this could be relaxed). Finally, *Λ* represents the force of infection on susceptible individuals during the transient phase, and can take different forms depending on the transmission mode of the parasite. For example, for a parasite that undergoes direct transmission within the cohort of hosts, *Λ* = *βI* (where *β* is the standard *per capita* transmission rate). However, for a parasite that infects from a pre-existing environmental reservoir, *Λ* will simply be a constant, reflecting the number of infectious stages in the environment encountered per unit time. Given this framework, the dynamics of hosts that successfully arrive at the recipient location (total: *A*; infected: *A*_I_) is given by3.3
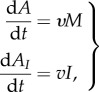
such that the total number of individuals arriving 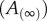 and number of infected individuals arriving 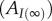 is3.4
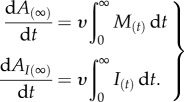


**Figure 3.**
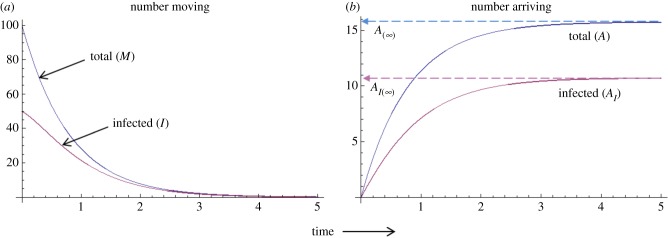
Dynamics of the total number of hosts and the number of infected ones during the transient moving phase as predicted from a mathematical model, assuming parasite transmission from the environment. (*a*) Total number of individuals (*M*) and number of infected individuals (*I*) undergoing transient movement through time. (*b*) Cumulative total number of individuals (*A*) and number of infected individuals arriving at the destination location through time (*A*_I_). We emphasize that this figure is for illustrative purposes only, created using arbitrary parameter values that do not relate to values from any particular empirical system (*d* = 1, *α* = 0.1, *Λ* = 1, *σ* = 0.1, *υ* = 0.2). (Online version in colour.)

Example dynamics for this model are shown in figure 3. Using this general framework, models can be developed that are tailored to the dynamics of specific host-parasite systems while meeting logistical constraints or data limitations. We emphasize that we do not aim here to provide a comprehensive analysis of the dynamical properties of this model, which is beyond the scope of this review. Instead, we present this framework to clarify the occurrence and connection of the various processes that affect infection spread during host transience.

Importantly, the parameters in this framework are likely to be influenced in different ways by host (*H*), parasite (*P*) and environmental (*E*) factors, and any interactions between them. As such, these parameters should be considered as functions, depending on *H*, *P* and *E*; for example,3.5
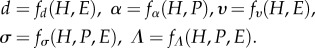
We argue that closer attention to each of these functions and, ideally, parameterizing (at least some of) the host, parasite and environmental dependencies within them will lead to a clearer and more mechanistic understanding of spatial host and infection dynamics than currently exists. In the following sections, we consider existing empirical evidence for these dependencies, and highlight gaps where further information is required.

## 4. Factors influencing transient phase infection dynamics

### Recovery (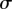) and relation to host arrival rate (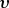)

(a)

Recovery from infections during host transience acts to decouple infection spread from host movement. As a consequence, so-called ‘structural delay effects’ [47], whereby parasite circulation predominantly occurs within resident locations, may occur even in host networks highly connected by movement. As a given time period (on average 1/*σ* time units in our framework) is required before recovery occurs [11], rates of recovery during transience depend fundamentally on the amount of time the hosts spend in transience (on average, 1/*υ* time units). The duration of transience is, at least in part, related to the linear distance travelled, and so simpler models may account for variation in recovery rates by considering differences in movement distances. Growing empirical evidence of infection recovery during long-distance seasonal migrations (electronic supplementary material, table S2) [7] suggests that decoupling effects of host recovery are particularly pronounced with longer linear distances. Substantial variation in the direction and velocity of intergroup movements can also occur within populations [49], so in many cases the time that hosts spend in transience may not correspond to the linear distance travelled. Characterizing variation in movement trajectories may therefore be important for parameterizing recovery rates. Even if the time that hosts spend in transience is, on average, longer than the infectious period, outlying cases of rapid movement or longer persistence of infection may sustain infection spread between resident locations. Thus, the degree of overlap in the variation in transient phase duration and infectious period should more accurately estimate rates of spread throughout spatial host networks.

Factors related to hosts and the environment that affect the time that hosts spend in transience may influence rates of spread. For example, behavioural responses to mitigate risks and costs of infection are well documented in wildlife, and can be manifested through changes in host movement patterns [48]. Landscape structure can also influence the duration of host transience with implications for infection spread [49]. Behavioural and landscape effects on host movement can be captured in our framework by allowing arrival rates (*υ*) to vary with infection loads and/or the presence of habitat features in the movement path.

As most local movements between nearby resident locations are probably too brief for infection recovery to occur, infection spread may be better predicted by transmission during host transience or by characteristics of resident locations (e.g. infection status [21], population size [1], spatial arrangement [13]). Recovery should not be completely disregarded for local dynamics, however. Abrupt changes in abiotic conditions that often occur when entering transience could result in rapid recovery events, for example, when fish move through saline waters [30,50]. Livestock lose ectoparasites during daily ranging movements between woodlands (favourable for ticks) and pasture (unfavourable for ticks), which modelling suggests can modulate infection prevalence in the broader population (figure 2*c*) [41].

### (b) Host mortality (background, *d*, or parasite-induced, *α*)

Mortality of hosts during transience clearly will affect the number of hosts that arrive (*A*). However, if infected hosts are differentially affected (via, for example, increased pathogenic effects (*α*) during movement) host mortality during transience will also affect the proportion of immigrants that carry infections to the destination (*A*_I_/*A*). This process may therefore inhibit parasite persistence through reductions in infection spread and reductions in susceptible hosts available for infection in recipient locations. Experimental work supports the hypothesis that infection-induced mortality is a mechanism underlying observed decreases in protozoal infections with distance migrated by monarch butterflies (*Danaus plexippus*, figure 2*b*) [51]. Immunological factors should play a role in this process. Some species balance the energetic costs of prolonged movement with immunosuppression [52], which clearly increases infection risk, and probably mortality, during host transience. Alternatively, adaptations that enhance immune function during periods of travel, particularly tolerance responses that aid host survival without resulting in parasite clearance [53], could facilitate infection spread. Such adaptations are evidenced by migratory birds that experience immune activation when preparing to migrate (figure 2*d*) [42] and by larger immune defence organs of migratory versus non-migratory bird species [54].

In addition to host-related factors, both parasite-related factors (rate of host exploitation) and environmental conditions may also affect infection-induced (*α*) and background (*d*) mortality rates of moving hosts at both local and regional scales. Traversing habitats with unfavourable conditions (e.g. extreme temperatures) or high densities of predators could drive host deaths during transience, irrespective of the distance travelled. Similarly, infections from highly virulent parasites acquired within source locations could conceivably compromise host health to an extent that even modest energy expenditures during local movement could cause death in transit.

### (c) Force of infection (*Λ*)

In contrast with recovery and mortality, transmission during host transience (either among moving hosts, at *per capita* rate *β*, or from the environment, at rate *Λ*) generally facilitates infection spread among host networks. This process therefore strengthens the link between infection spread and host movement, but weakens the link between spread and prevalence in source resident locations. As gains in infection are contingent on susceptible hosts encountering infective stages, either from other infected hosts or in the environment, we expect that the rate of acquisition of new infections during host transience is most dependent on parasite transmission mode, the habitats traversed in the transient phase, and the grouping patterns of moving hosts. For environmentally transmitted parasites, acquisition of infection during host transience results when moving hosts traverse habitats supporting infective stages. Primates typically acquire helminth infections during daily ranging [55], and modelling suggests that transmission during local ranging of primate individuals can allow parasites to invade and expand in their populations [56]. Acquisition of infection during host transience may also explain the apparent importance of inter-burrow movement of pygmy blue-tongued lizards (*Tiliqua adelaidensis*) for local infection spread (figure 2*g*) [9].

At broader scales, the epidemiological relevance of transmission during host transience is well illustrated by seasonal migrations of saiga (*Saiga tatarica*) [8]. Saiga acquire infections while moving through pastures with sheep faecal matter that harbour infective nematode stages (figure 2*f*). For nematodes therefore, spatial spread is contingent on transmission in saiga during the transient phase rather than transmission within resident locations [8], emphasizing again how habitats traversed during host transience can factor into spatial infection dynamics. Energy expenditure and immunosuppression during regional movements may amplify transmission by activating infections from dormant parasite stages. Outbreaks of latent bacterial (*Borrelia garinii*) infections occurred in redwing thrushes (*Turdus iliacus*) when migratory restlessness was induced (figure 2*h*) [44]. Activation of latent fungal infections has also been reported in natterjack toads (*Epidalea calamita*) when moving from terrestrial to aquatic habitats [57].

For vector-borne infections, transmission during host transience depends on moving hosts encountering habitats favourable for vectors as well as the parasites they harbour. Daily movements of humans can increase time in habitats harbouring mosquito-borne dengue virus [58] and result in spatial patterns of infection risk that diverge from those predicted by abundance of mosquitoes in households [58]. These findings support the hypothesis that exposure during host transience (captured by the force of infection parameter, *Λ*, in our framework) may decrease the influence of resident locations on patterns of infection spread.

Grouped travel probably enhances transmission of directly transmitted parasites among moving hosts. Studies of shoaling movements in fish demonstrate that parasitic infections can be transmitted in travelling groups [59]. Documentation of avian influenza virus transmission during stopovers along bird migration routes lends further support for the potential of grouped travel to promote transmission during host transience (figure 2*i*) [45]. Alternatively, assortative grouping patterns could inhibit transmission among transient hosts (i.e. migratory allopatry). Migration by juvenile pink salmon (*Oncorhynchus gorbuscha*) prevents acquisition of infection through separation from infective adults (figure 2*e*) [43]. This case is represented in our framework through a *β* parameter equal to zero and would result in structural trapping of infection to locations occupied by adult hosts.

## 5. Future direction

This review highlights that obtaining field data on infection dynamics during the transient phase of movement present a key challenge to understanding the mechanistic links of host movement and infection spread. Owing to the recent innovations of tracking and computational technology that permit detailed individual-based tracking of wildlife systems [38], we argue that collection of such data is now feasible for some wildlife systems. Utilization of automated image-based tracking methods [60] allows ecologists to characterize at high resolutions the behavioural patterns of infected and uninfected hosts in controlled environments that mimic transient phases. These approaches also provide the opportunity to quantify effects of host grouping on transmission during transient phases. A key advantage of these experimental approaches is the feasibility of monitoring changes in infections in individual hosts at fine temporal scales, which can be directly linked to environmental conditions and host behaviours. Nevertheless, owing to costs and logistical constraints, image-based tracking is typically performed in small experimental units. Distinguishing departure, transience and arrival in small units can be problematic. Future effort can be made to develop larger experimental tracking systems, such as mesocosms, capable of capturing all phases of host movement and infection spread.

The radio-tracking and GPS studies highlighted above [19,39,61] are strong initial attempts at directly quantifying transient phase host movements in the wild. Future work can improve on these approaches by combining movement paths with individual infection data at multiple points during transience. Doing so can better identify factors that decouple rates of infection spread from linear host movement assumed in conventional models, which might resolve unexpected and inconsistent findings of prior work [9,19]. For organisms that cannot be feasibly surveyed for infection during transient phases, biologging devices may be developed that remotely assay infection status of moving hosts in the wild. This could also be done indirectly. For example, as immune function in ectothermic animals is strongly linked to body temperature, fitting migratory ectotherms such as amphibians and snakes with temperature sensors may provide insights into how host susceptibility varies during periods of movement. For larger-bodied mammals, GPS devices combined with accelerometers can identify critical periods of movement during which increased energy expenditure poses heightened infection risk [38].

Considering the importance of the structure and abiotic conditions of the habitat matrix surrounding resident locations for transient phase infection dynamics, approaches used by landscape epidemiologists can benefit spatial network models of infection spread. Landscape epidemiologists apply environmental data from satellite imagery to identify the habitats in which diseases proliferate. Integration of habitat data into metapopulation models has been carried out extensively [49,62,63], but models have typically only considered effects of habitat on host movement. Future work can advance by considering realistic effects that differential quality of habitats in the matrix has on transmission and host recovery during periods of movement [17,18]. Additionally, the coarse resolution of much environmental data used in landscape epidemiological studies limits the utility of these data to regional movements such as migrations and dispersal. Local scale heterogeneities in external conditions (e.g. moisture levels [64], vegetation cover [65], temperature [64,66], predation risk [67]) are known to affect infection risk and prevalence and may also affect host infections during local movements. Experiments that manipulate habitat can complement landscape ecological approaches by testing how movement through the habitat matrix alters courses of infection within hosts. In addition, field and experimental data on the abundance and persistence of parasite infective stages and/or infection vectors in the habitat matrix can inform parameterization of rates of environmental transmission in transient hosts. Theoretical work has begun to use these types of data to explore infection dynamics in single locations [68], and our framework can guide spatially explicit extensions of these models that distinguish environmental transmission rates at each phase of host movement. Finally, human alteration of habitats comprising host networks, while posing various potentially detrimental consequences for population viability, may afford natural experiments for testing the abiotic factors involved in transience phase infection dynamics. Satterfield *et al*. [69] were able to use human-mediated amplification of exotic milkweed (*Asclepias curassavica*) in the USA, a preferred breeding and nutrient resource of monarch butterflies, to model how loss of migratory behaviour in monarch populations caused by year-round resource availability altered population-level infection dynamics. Human activities that alter the habitats spanning spatial host networks may allow ecologists to measure the effects of habitat structure, temperature, moisture and other abiotic variables on infection in transient hosts. Such data would enhance the ability to predict patterns of disease spread amid environmental change.

## 6. Conclusion

Identification of relevant biological processes is the first step in building mechanistic models of ecological dynamics. With an explicit transient phase, our conceptual framework unpacks infection spread into its constituent biological processes: transmission, infection recovery and infection-induced mortality. In so doing, our framework links patterns of infection spread described by existing spatial models to specific mechanisms that otherwise are hidden in their assumptions. While our framework can be simplified as needed, evidence of these processes from the empirical studies reviewed here provides a strong rationale for building this added complexity into disease models. Owing to technological developments, movement ecology is experiencing an exciting renaissance of big data that is affording new insights in the mechanisms driving animal movements as well as their ecological consequences. These developments provide equally exciting opportunities for disease ecologists to advance our understanding of the consequences of host movement for infection spread, the factors that determine those consequences, and how to model spatial infection dynamics.

## Supplementary Material

Table S1

## Supplementary Material

Table S2

## Supplementary Material

Captions and references for supplementary tables

## Data accessibility

This article has no additional data.
