# Supplementary material for: Infections on the move: how transient phases of host movement influence disease spread
Source: Proc Biol Sci. 2017 Dec 20;284(1869):20171807. doi: 10.1098/rspb.2017.1807 (PMC5745403; doi:10.1098/rspb.2017.1807)
Supplement: Table S1 [file rspb20171807supp1.pdf]

model class	Reference	Host system	Parasite system	movement phases	movement factors	movement data	infection spread processes	infection spread factors	infection data
proximity-based metapopulation	Ajelli et al. 2010*	humans	Influenza virus	-	-	census data airport records	force of infection	node size and proximity (gravity)	government records
	Balcon et al. 2009	humans	Influenza virus	-	-	govn't records airport records	-	node size and proximity (gravity)	government records
	Charu et al. 2017	humans	Influenza virus	-		census data govn't records		node size and proximity (gravity)	physician recrods
	Davis et al. 2008	great gerbils *(Rhombomys opimus)*	plague *(Yersinia pestis)*	-	-	field data (mark-recapture)	-	inter-node distance (percolation)	-
	Laperrière et al. 2016	great gerbils *(Rhombomys opimus)*	fleas *(Xenopsylla gerbilli)*	-	-	field data (mark-recapture)	-	inter-node distance	-
	Gurarie and Seto 2009	humans	blood flukes *(Schistosoma japonicum)*	-	-	-	-	node size and proximity (gravity)	-
	Remais et al. 2010	humans	blood flukes *(Schistosoma japonicum)*	-	-	-	-	proximity to streams landscape structure	-
	Xia et al. 2004	humans	measles *(Morbillivirus)*	-	-	-	-	node size and proximity (gravity)	government records
kernel-based metapopulation	Becker and Hall, 2016	non-specific	non-specific	D-A	-	-	-	habitat quality, provisioning	-
	Craft et al. 2011*	Serengheti lions *(Panthero leo)*	canine distemper virus *(Morbillivirus)*	D-A	host phenotype (nomad vs resident)	field data (GPS)	-	infectious period	-
	Cross et al. 2005	non-specific	non-specific	D-A	none	-	-	-	-
	Dalziel et al. 2013	humans	non-specific	D-A	host identity	census data		-	-
	Fulford et al. 2002	brushtail possums *(Trichosurus vulpecula)*	bovine tuberculosis *(Mycobacterium bovis)*	D-A	age, migrant mortality	-	-	-	-
	Gog et al. 2002	non-specific	non-specific	D-A	none	-	-	-	-
	Green et al. 2006	livestock	foot-and-mouth disease *(Apthae epizooticae)*	D-A	individual identity	Govn't records	-	-	Govn't records
	Harding et al. 2012	non-specific	non-specific	D-A	-	-	-	allee effects	-
	Hess et al. 1996	non-specific	non-specific	D-A	node density	-	-	-	-

	Leach et al., 2016	non-specific	non-specific	D-A	node density	-	-	habitat quality	
	McCallum and Dobson 2002	non-specific	non-specific	D-A	-	-	-	-	-
	Park, 2012	non-specific	non-specific	D-A	-	-	-	-	-
	Russel et al. 2006	raccons *(Procyon lotor)*	rabies *(Lyssavirus)*	D-A	node density	-	-	-	-
	Colizza et al., 2008	humans	non-specific	D-A	traffic, node density	census data airline records	-	group size	-
Coupled metapopulation	Jesse et al. 2008	non-specific	non-specific	D-A	-		-		-
	Jesse and Hesterbeek 2011	non-specific	non-specific	D-A	preferred movement distance	-	-	-	-
individual-based spatial network	Ajelli et al. 2010*	humans	Influenza virus	D-A	host identity	census data	force of infection	distance	-
	Aleta et al. 2017	humans	Influenza-like virus	D-A	-	-	-	-	-
	Bonnel et al. 2010	red colobus monkeys *(Procolobus rufomitratus)*	microparasites (non-specific)	D-T-A	Food resource distribution	-	force of infection (direct transmission)	node proximity	-
	Craft et al. 2011*	Serengheti lions *(Panthero leo)*	canine distemper virus *(Morbillivirus)*	D-T-A	host phenotype (nomad vs resident)	field data (GPS)	-	infectious period	-
	Keeling et al. 2010	humans	multiple	D-A	host phenotype (commuter/non-commuter)	census data	-	time of day	Govn't records
	Meloni et al. 2011	humans	non-specific	D-A	infection risk in patches	-	-	-	-
	Riley and Ferguson 2006	humans	smallpox	D-A	host identity	census data	force of infection	-	-
	Springer et al. 2017	*sifakas*	*Chryptosporidium*	D-A	host identity	field data (GPS)	force of infection (environmental transmission)	range overlap	-
	Tracey et al. 2014	bobcats	Feline immunodeficiency virus	D-A	host identity landscape structure	field data (GPS)	-	-	-
