# Supplementary material for: Infections on the move: how transient phases of host movement influence disease spread
Source: Proc Biol Sci. 2017 Dec 20;284(1869):20171807. doi: 10.1098/rspb.2017.1807 (PMC5745403; doi:10.1098/rspb.2017.1807)
Supplement: Table S2 [file rspb20171807supp2.pdf]

Movement type	Method	movement phases	parasite	host	outcome	possible transient phase processes	possible transient phase factors	reference
dispersal	genetics (gene flow)	D - A	freshwater trematodes	long-finned eel *(A. dieffenbachii)*	overlapping genetic structures	none cited	none cited	Blasco-costa et al. 2012
	genetics (gene flow)	D - A	marine trematode *(Gymnophallus choledochus)*	irds (e.g. ducks, gulls, wader	overlapping genetic structures	none cited	none cited	Feis et al. 2015
	genetics (assignment tests)	D - A	black-tailed prairie dog *Cynomys ludovicianus*	fleas *Oropsylla hirsuta*	unrelated genetic structures	none cited	none cited	Jones and Britten 2010
	genetics (viral gene flow)	D - A	vampire bats *Desmodus rotundus*	rabies virus *Lyssavirus, Rabodviridae*	overlapping genetic structures	none cited	landscape structure	Streiker et al. 2016
local	mark-recapture	D - A	Swallow bugs *(Oeciacus vicarius)*	cliff swallows *(Petrochelidon pyrrhonota)*	increased prevalence	none cited	none cited	Brown and Brown 2004
	GPS tracking	D - T - A	canine distemper virus *(Morbillivirus)*	serengheti lions *(Panthera leo)*	no effect by nomads	recovery	host grouping, movement behaviour	Craft et al. 2011
	mark-recapture	D - A	nematodes *(Pharyngodon wandillahensis)*	pygmy blue-tongued lizard *(Tiliqua adelaidensis)*	increased transmission	infection forcing (environmental transmission)	habitat	Fenner et al. 2011
migration	Citizen Science surveys	D - T - A	protozoa *(Ophryocystis elektroscirrha)*	monarch butterfly *(Danaeus plexxipus)*	decreased prevalence	infection forcing host mortality	host grouping, habitat	Altizer et al. 2000, Bartel et al. 2011
	remote tracking (radio telemetry)	D - T	chytrid fungus *(Batrachochytrium dendrobatidis)*	common toad *(Bufo bufo)*	decreased prevalence	recovery	habitat	Daversa et al. unpublished
	mark-recapture	D - A	avian influenza virus	mallard ducks *(Anas platyrhyncos)*	increased prevalence	none cited	none cited	van Dijk et al. 2014
	Citizen Science surveys	D - T - A	bacteria *(Mycoplasma gallisepticum)*	house finches *(Carpodacus mexicanus)*	increased prevalence	none cited	none cited	Hosseini et al. 2011
	mark-recapture	D- T - A	avian influenza virus	pink-footed geese *(Anser brachyrhynchus)*	decreased prevalence	recovery	host grouping (allopatry), habitat	Hoye et al. 2011
	Field surveys	T	sea lice *(Lepeophtheirus salmonis, Caligus clemensi)*	pink salmon *(Oncorhynchus gorbuscha)*	decreased prevalence	no infections present	host grouping (allopatry)	Krkosek et al. 2007
	remote tracking (GPS)	D - A	ticks *(Ixodes ricinus)*	red deer *(Cervus elaphus)*	decreased prevalence	recovery	habitat (elevation)	Mysterud et al. 2016
	mark-recapture	D - A	trematodes	galaaxid fish *(Galaxia spp.)*	decreased prevalence	recovery	habitat (water salinity)	Poulin. et al. 2012
