# Supplementary material for: Infections on the move: how transient phases of host movement influence disease spread
Source: Proc Biol Sci. 2017 Dec 20;284(1869):20171807. doi: 10.1098/rspb.2017.1807 (PMC5745403; doi:10.1098/rspb.2017.1807)
Supplement: Captions and references for supplementary tables [file rspb20171807supp3.docx]

Supplementary Material for:

*Infections on the move: How transient phases of host movement influence disease spread*

DR Daversa, A Fenton, AI Dell, TWJ Garner, and A Manica

Proceedings of the Royal Society B

DOI: 10.1098/rspb. 2017.1807

**Table S1.** A list of theoretical studies to use various classes of spatial network models as described in Fig. 1. Movement phase identifies which of the three phases of movement as defined by dispersal ecologists [1,2] that the model considers (D = departure, T = transience and A = arrival). * Studies used multiple classes of model and hence are listed twice in the table.

**Table S2.** A list of empirical studies to evaluate links between infection spread and host movement using genetic, mark-recapture or remote tracking approaches. Movement phase identifies which of the three phases of movement for which the studies obtained data (D – departure, T – transience, A – arrival). For a more comprehensive list of relevant studies to use genetic approaches, we refer the reader to [3].

1. Clobert J, Le Galliard J-F, Cote J, Meylan S, Massot M. 2009 Informed dispersal, heterogeneity in animal dispersal syndromes and the dynamics of spatially structured populations. *Ecol. Lett.* **12**, 197–209. (doi:10.1111/j.1461-0248.2008.01267.x)

2. Travis JMJ *et al.* 2012 Modelling dispersal: an eco-evolutionary framework incorporating emigration, movement, settlement behaviour and the multiple costs involved. *Methods Ecol. Evol.* **3**, 628–641. (doi:10.1111/j.2041-210X.2012.00193.x)

3. Mazé-Guilmo E, Blanchet S, McCoy KD, Loot G. 2016 Host dispersal as the driver of parasite genetic structure: a paradigm lost? *Ecol. Lett.* , 336–347. (doi:10.1111/ele.12564)

**Table S1 References**

Ajelli M, Gonçalves B, Balcan D, Colizza V, Hu H, Ramasco JJ, Merler S, Vespignani A. 2010 Comparing large-scale computational approaches to epidemic modeling: agent-based versus structured metapopulation models. *BMC Infect. Dis.* **10**, 190.

Aleta A, Hisi ANS, Meloni S, Poletto C, Colizza V, Moreno Y. 2017 Human mobility networks and persistence of rapidly mutating pathogens. *R. Soc. Open Sci.* **4**, 160914. (doi:10.1098/rsos.160914)

Altizer S, Bartel R, Han BA. 2011 Animal migration and infectious disease risk. *Science* **331**, 296–302. (doi:10.1126/science.1194694)

Balcan D *et al.* 2009 Seasonal transmission potential and activity peaks of the new influenza A (H1N1): a Monte Carlo likelihood analysis based on human mobility. *BMC Med.* **7**, 45. (doi:10.1186/1741-7015-7-45)

Bartel RA, Oberhauser KS, De Roode JC, Altizer SM. 2011 Monarch butterfly migration and parasite transmission in eastern North America. *Ecology* **92**, 342–351.

Becker DJ, Hall RJ. 2014 Too much of a good thing: resource provisioning alters infectious disease dynamics in wildlife. *Biol. Lett.* **10**, 20140309–20140309. (doi:10.1098/rsbl.2014.0309)

Bonnell TR, Sengupta RR, Chapman CA, Goldberg TL. 2010 An agent-based model of red colobus resources and disease dynamics implicates key resource sites as hot spots of disease transmission. *Ecol. Model.* **221**, 2491–2500. (doi:10.1016/j.ecolmodel.2010.07.020)

Charu V, Zeger S, Gog J, Bjørnstad ON, Kissler S, Simonsen L, Grenfell BT, Viboud C. 2017 Human mobility and the spatial transmission of influenza in the United States. *PLOS Comput. Biol.* **13**, e1005382. (doi:10.1371/journal.pcbi.1005382)

Colizza V, Vespignani A. 2008 Epidemic modeling in metapopulation systems with heterogeneous coupling pattern: Theory and simulations. *J. Theor. Biol.* **251**, 450–467. (doi:10.1016/j.jtbi.2007.11.028)

Craft ME, Volz E, Packer C, Meyers LA. 2011 Disease transmission in territorial populations: the small-world network of Serengeti lions. *J. R. Soc. Interface* **8**, 776–786. (doi:10.1098/rsif.2010.0511)

Cross PC, Lloyd-Smith JO, Johnson PLF, Getz WM. 2005 Dueling timescales of host movement and disease recovery determine invasion of disease in structured populations. *Ecol. Lett.* **8**, 587–595. (doi:10.1111/j.1461-0248.2005.00760.x)

Dalziel BD, Pourbohloul B, Ellner SP. 2013 Human mobility patterns predict divergent epidemic dynamics among cities. *Proc. R. Soc. B Biol. Sci.* **280**, 20130763–20130763. (doi:10.1098/rspb.2013.0763)

Davis S, Trapman P, Leirs H, Begon M, Heesterbeek JAP. 2008 The abundance threshold for plague as a critical percolation phenomenon. *Nature* **454**, 634–637. (doi:10.1038/nature07053)

Fulford GR, Roberts MG, Heesterbeek JAP. 2002 The metapopulation dynamics of an infectious disease: Tuberculosis in possums. *Theor. Popul. Biol.* **61**, 15–29. (doi:10.1006/tpbi.2001.1553)

Gog J, Woodroffe R, Swinton J. 2002 Disease in endangered metapopulations: the importance of alternative hosts. *Proc. R. Soc. B Biol. Sci.* **269**, 671–676. (doi:10.1098/rspb.2001.1667)

Green D., Kiss I., Kao R. 2006 Modelling the initial spread of foot-and-mouth disease through animal movements. *Proc. R. Soc. B Biol. Sci.* **273**, 2729–2735. (doi:10.1098/rspb.2006.3648)

Gurarie D, Seto EYW. 2009 Connectivity sustains disease transmission in environments with low potential for endemicity: modelling schistosomiasis with hydrologic and social connectivities. *J. R. Soc. Interface* **6**, 495–508. (doi:10.1098/rsif.2008.0265)

Harding KC, Begon M, Eriksson A, Wennberg B. 2012 Increased migration in host–pathogen metapopulations can cause host extinction. *J. Theor. Biol.* **298**, 1–7. (doi:10.1016/j.jtbi.2011.12.009)

Hess G. 1996 Disease in metapopulation models: implications for conservation. *Ecology* **77**, 1617. (doi:10.2307/2265556)

Jesse M, Ezanno P, Davis S, Heesterbeek JAP. 2008 A fully coupled, mechanistic model for infectious disease dynamics in a metapopulation: Movement and epidemic duration. *J. Theor. Biol.* **254**, 331–338. (doi:10.1016/j.jtbi.2008.05.038)

Jesse M, Heesterbeek H. 2011 Divide and conquer? Persistence of infectious agents in spatial metapopulations of hosts. *J. Theor. Biol.* **275**, 12–20. (doi:10.1016/j.jtbi.2011.01.032)

Keeling MJ, Danon L, Vernon MC, House TA. 2010 Individual identity and movement networks for disease metapopulations. *Proc. Natl. Acad. Sci.* **107**, 8866–8870. (doi:10.1073/pnas.1000416107)

Laperrière V, Brugger K, Rubel F. 2016 Cross-scale modeling of a vector-borne disease, from the individual to the metapopulation: The seasonal dynamics of sylvatic plague in Kazakhstan. *Ecol. Model.* **342**, 34–48. (doi:10.1016/j.ecolmodel.2016.09.023)

Leach CB, Webb CT, Cross PC. 2016 When environmentally persistent pathogens transform good habitat into ecological traps. *R. Soc. Open Sci.* **3**, 160051. (doi:10.1098/rsos.160051)McCallum H, Dobson A. 2002 Disease, habitat fragmentation and conservation. *Proc. R. Soc. Lond. B Biol. Sci.* **269**, 2041–2049. (doi:10.1098/rspb.2002.2079)

Meloni S, Perra N, Arenas A, Gómez S, Moreno Y, Vespignani A. 2011 Modeling human mobility responses to the large-scale spreading of infectious diseases. *Sci. Rep.* **1**, 1. (doi:10.1038/srep00062)

Park AW. 2012 Infectious disease in animal metapopulations: the importance of environmental transmission: Infectious disease in animal metapopulations. *Ecol. Evol.* **2**, 1398–1407. (doi:10.1002/ece3.257)

Remais J, Akullian A, Ding L, Seto E. 2010 Analytical methods for quantifying environmental connectivity for the control and surveillance of infectious disease spread. *J. R. Soc. Interface* **7**, 1181–1193. (doi:10.1098/rsif.2009.0523)

Riley S, Ferguson NM. 2006 Smallpox transmission and control: spatial dynamics in Great Britain. *Proc. Natl. Acad. Sci.* **103**, 12637–12642.

Russell CA, Real LA, Smith DL. 2006 Spatial control of rabies on heterogeneous landscapes. *PLoS One* **1**, e27.

Springer A, Kappeler PM, Nunn CL. 2017 Dynamic vs. static social networks in models of parasite transmission: predicting *Cryptosporidium* spread in wild lemurs. *J. Anim. Ecol.* **86**, 419–433. (doi:10.1111/1365-2656.12617)

Tracey JA, Bevins SN, VandeWoude S, Crooks KR. 2014 An agent-based movement model to assess the impact of landscape fragmentation on disease transmission. *Ecosphere* **5**, art119.

Xia Y, Bjørnstad ON, Grenfell BT. 2004 Measles metapopulation dynamics: A gravity model for epidemiological coupling and dynamics. *Am. Nat.* **164**, 267–281. (doi:10.1086/422341)

**Table S2 References**

Altizer S, Bartel R, Han BA. 2011 Animal migration and infectious disease risk. *Science* **331**, 296–302. (doi:10.1126/science.1194694)

Bartel RA, Oberhauser KS, De Roode JC, Altizer SM. 2011 Monarch butterfly migration and parasite transmission in eastern North America. *Ecology* **92**, 342–351.

Blasco-Costa I, Waters JM, Poulin R. 2012 Swimming against the current: genetic structure, host mobility and the drift paradox in trematode parasites. *Mol. Ecol.* **21**, 207–217. (doi:10.1111/j.1365-294X.2011.05374.x)

Brown CR, Brown MB. 2004 Empirical measurement of parasite transmission between groups in a colonial bird. *Ecology* **85**, 1619–1626.

Craft ME, Volz E, Packer C, Meyers LA. 2011 Disease transmission in territorial populations: the small-world network of Serengeti lions. *J. R. Soc. Interface* **8**, 776–786. (doi:10.1098/rsif.2010.0511)

van Dijk JGB, Hoye BJ, Verhagen JH, Nolet BA, Fouchier RAM, Klaassen M. 2014 Juveniles and migrants as drivers for seasonal epizootics of avian influenza virus. *J. Anim. Ecol.* **83**, 266–275. (doi:10.1111/1365-2656.12131)

Feis M, Thieltges D, Olsen J, de Montaudouin X, Jensen K, Bazaïri H, Culloty S, Luttikhuizen P. 2015 The most vagile host as the main determinant of population connectivity in marine macroparasites. *Mar. Ecol. Prog. Ser.* **520**, 85–99. (doi:10.3354/meps11096)

Fenner AL, Godfrey SS, Michael Bull C. 2011 Using social networks to deduce whether residents or dispersers spread parasites in a lizard population: social networks and parasite transmission. *J. Anim. Ecol.* **80**, 835–843. (doi:10.1111/j.1365-2656.2011.01825.x)

Hosseini PR, Dhondt AA, Dobson AP. 2006 Spatial spread of an emerging infectious disease: conjunctivitis in house finches. *Ecology* **87**, 3037–3046.

Hoye BJ, Munster VJ, Nishiura H, Fouchier RAM, Madsen J, Klaassen M. 2011 Reconstructing an annual cycle of interaction: natural infection and antibody dynamics to avian influenza along a migratory flyway. *Oikos* **120**, 748–755. (doi:10.1111/j.1600-0706.2010.18961.x)

Jones PH, Britten HB. 2010 The absence of concordant population genetic structure in the black-tailed prairie dog and the flea, *Oropsylla hirsuta*, with implications for the spread of Yersinia pestis: host-vector population genetic structure. *Mol. Ecol.* **19**, 2038–2049. (doi:10.1111/j.1365-294X.2010.04634.x)

Krkosek M, Gottesfeld A, Proctor B, Rolston D, Carr-Harris C, Lewis MA. 2007 Effects of host migration, diversity and aquaculture on sea lice threats to Pacific salmon populations. *Proc. R. Soc. B Biol. Sci.* **274**, 3141–3149. (doi:10.1098/rspb.2007.1122)

Mysterud A, Qviller L, Meisingset EL, Viljugrein H. 2016 Parasite load and seasonal migration in red deer. *Oecologia* **180**, 401–407. (doi:10.1007/s00442-015-3465-5)

Poulin R, Closs GP, Lill AWT, Hicks AS, Herrmann KK, Kelly DW. 2012 Migration as an escape from parasitism in New Zealand galaxiid fishes. *Oecologia* **169**, 955–963. (doi:10.1007/s00442-012-2251-x)

Streicker DG *et al.* 2016 Host–pathogen evolutionary signatures reveal dynamics and future invasions of vampire bat rabies. *Proc. Natl. Acad. Sci.* **113**, 10926–10931. (doi:10.1073/pnas.1606587113)
